# Recent Technologies on 2D and 3D Imaging Flow Cytometry

**DOI:** 10.3390/cells13242073

**Published:** 2024-12-16

**Authors:** Masashi Ugawa, Sadao Ota

**Affiliations:** 1 Research Center for Advanced Science and Technology, University of Tokyo, Tokyo 153-8904, Japan; 2 Department of Bioengineering and Therapeutic Sciences, University of California, San Francisco, CA 94143, USA; 3 ThinkCyte, Inc., Tokyo 113-0033, Japan

**Keywords:** imaging flow cytometry, flow cytometry, cell sorting, cytometry

## Abstract

Imaging flow cytometry is a technology that performs microscopy image analysis of cells within flow cytometry and allows high-throughput, high-content cell analysis based on their intracellular molecular distribution and/or cellular morphology. While the technology has been available for a couple of decades, it has recently gained significant attention as technical limitations for higher throughput, sorting capability, and additional imaging dimensions have been overcome with various approaches. These evolutions have enabled imaging flow cytometry to offer a variety of solutions for life science and medicine that are not possible with conventional flow cytometry or microscopy-based screening. It is anticipated that the extent of applications will expand in the upcoming years as the technology becomes more accessible through dissemination. In this review, we will cover the technical advances that have led to this new generation of imaging flow cytometry, focusing on the advantages and limitations of each technique.

## 1. Introduction

Single-cell analysis technologies have developed rapidly in recent decades, enabling high-resolution cell analysis on a large scale [1,2,3,4]. This also holds true in the field of flow cytometry, where imaging technologies have been combined with high-throughput flow-cytometric sampling to perform high-content optical analysis of each individual cell on a large scale [5,6,7,8,9,10,11]. Such technology, called imaging flow cytometry (IFC), has extensively advanced over the past decade.

Despite the recent-growing trend in IFC, the concept of IFC dates back to the 1980s, only a couple of years after the invention of the conventional state-of-the-art flow cytometers that perform analysis based on optical intensity measurements [12,13,14]. The original purpose of IFC was to reduce false negatives and false positives from its acquisition by obtaining more information from a single event (i.e., the detected signal from a cell or a particle). Still, these technologies for acquiring high-dimensional data at high-throughputs and performing computation on large-scale data were not mature enough. Therefore, it was not until decades later when this idea to perform high-throughput, high-dimensional analysis was realized in a practical implementation [15].

Aside from the technical difficulty of IFC at the time, conventional flow cytometry technologies have matured enough to become a necessity in biological and medical laboratories [16,17,18,19,20]. This is because for many biologically valuable analyses, the presence or absence of a protein or other biological molecules inside or on a surface of cells is a key parameter, and multiplexing these parameters using different fluorophores has enabled identifying a diverse range of cell populations. Furthermore, the sorting technology implemented with flow cytometers has allowed separation or isolation of cells based on such biomarker analysis, which is extremely powerful for their downstream analysis or reutilization [16,21].

Nevertheless, there are still analyses that conventional flow cytometry cannot perform, and IFC is gaining attention to address those shortcomings. For example, proteins can condensate or diffuse within a cell, and observing the difference in such behavior can be used to detect aberrant cells or monitor drug response [22,23,24]. This localization difference is difficult to detect by the fluorescence intensity measurement in conventional flow cytometry because the total number of the target molecules for each cell does not differ. In a similar fashion, observing co-localization of proteins also requires identification of the spatial localization of the proteins with fluorescence [25,26]. Additionally, well-known phenomena such as the different phases in mitosis are typically identified by observing the structure and orientation of cell organelles such as chromosomes [27]. In these cases where we know what molecules or organelles to target, fluorescence IFC can be an effective approach to study the hypothesized image features as intended. In contrast, there are cases where either (i) there are no effective staining markers available, (ii) requires multiple-steps of staining, (iii) demands substantial time and cost, or (iv) staining should be avoided to prevent chemical toxicity to the cells [28,29]. In such cases, label-free IFC is a powerful approach to distinguish cell types and conditions by observing cell shapes and subcellular features in detail [5,9,30,31,32].

Still, IFC has several caveats when compared to conventional flow cytometry. The most prominent downside is its throughput, or the number of cells that it can process in a unit amount of time. As IFC takes higher-dimensional data for each event or cell, the throughput is intrinsically slower compared to conventional flow cytometry. Commercial IFCs, at the highest end, work between 1000 cells/s to 15,000 cells/s [9], while current state-of-the-art flow cytometers can operate at over 20,000 cells/s [33]. Another downside of IFC is that the data acquired will become enormous compared to in flow cytometry. An IFC data acquisition for a population of cells can easily scale to gigabytes and, depending on the scale, even up to terabytes. This not only requires vast space of disk storage to save the data, but also requires computational resources to analyze the large-scale data. On top of that, analyses for IFC data are still less established than conventional flow cytometry and require user skills to extract meaningful information from the data [34]. Besides the limitation from speed and scale, IFC places more stringent demands on flow control because imaging requires the cells to flow through the focus of the imaging optics, which is in the range of a few to ten micrometers. This requirement is less severe in conventional flow cytometry because of the longer depth of focus of weakly focused beams in typical flow cytometers [16]. Consequently, it may be necessary to be aware that the degree of image focus can vary and incorporate this fact into data processing and analysis [35].

One question that arises when considering whether to use IFC is whether a standard microscopy system should be used instead of IFC, and indeed there are still many cases where standard microscopy is beneficial. First, standard microscopy systems are advantageous for routine analysis because they can image small cell populations without having to remove them from the container in which they were cultured. Furthermore, IFC requires the cells to be detached from substrates and dissociated in a suspension, inevitably changing the shape of the cells from their adherent state and causing a loss of positional and intercellular information. In addition, time-lapse imaging with IFC does not allow individual cells to be tracked over time as in the case of microscopy, and therefore, the change in cell shape cannot be observed for each cell.

Nevertheless, IFC has the advantage of having high throughput and the ability to measure and analyze large-scale cell populations, especially when the scale increases to millions of cells. Scaling up microscopy systems to this order of magnitude often requires automated exchange of vessels, which limits the overall throughput. In addition, the capability of high-throughput sorting based on imaging, which has become available in recent IFC [7,8,9,36], enables enrichment and isolation of cells, which would again be substantially limited by scale for microscopy systems. In this review, we will outline the technologies that have enabled high-throughput imaging and discuss prospects.

## 2. Technologies for IFC

### 2.1. Conventional Technology: ImageStream

**Figure 1:**
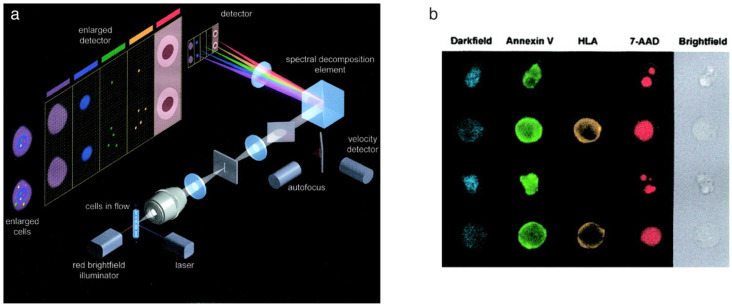
ImageStream. (**a**) Optical schematic. (**b**) Example images of cells obtained with ImageStream. Adapted from [15] with permission from John Wiley and Sons.

The first commercial imaging flow cytometer, ImageStream, was developed in the late 1990s and was the only commercially available IFC system for over a decade [5,15]. The key technology that made this IFC possible was the time-delay-and-integration charge-coupled device (TDI-CCD), which allowed imaging of flowing cells at low fluorescence intensity levels. This TDI-CCD acquires light at the pixels while electronically shifting the measurement as the cell moves so that the fluorescence signals from a single pixel on the cell image are integrated while the cell traverses the CCD array. Furthermore, a dichroic-filter stack allows multiple color channels to be imaged simultaneously on the same CCD with a total of 12 image channels for a single cell (Figure 1).

This commercially available system was tested for a variety of applications to explore its potential. As a biological cell analysis, the changes in cell shape during the cell cycle were investigated. This included one of the earliest attempts to analyze cell images using machine learning [37,38], soon after deep learning had proven powerful in image classification [39,40]. For more practical applications, medical diagnosis applying IFC on blood cells has been widely studied, including white blood cell differentiation [41], leukemia diagnosis [42], and blood quality assessment [43]. IFC was also tested in drug screening, where the efficacy of drug candidates for a parasite was analyzed by monitoring changes in the position of fluorescence within a cell [5].

While ImageStream has long pioneered the instrument market and applications for IFC, the technical limitations of ImageStream motivated new developments in the next generation of IFC technology. The first limitation is its throughput of 5000 cells/second at the most, which is significantly lower for practical acquisition, restricting the scale of analysis. The overlying reason for this is that it performs acquisition with a CCD, which has a slower data acquisition rate compared to PMTs used in flow cytometers. Another major limitation of ImageStream is that it lacks cell sorting functions, making it difficult to perform downstream analysis of the cells of interest after analyzing their image information. The last limitation, although not always an essential feature, is that it only provides 2D images and not 3D images; there are cases when analysis of 3D images becomes necessary, and this will be discussed in depth later in this review.

### 2.2. Two-Dimensional IFC

For about a decade, many technologies for performing a variety of 2D IFC have been reported and commercialized [6]. The most straightforward way to perform IFC is with a pixel-arrayed sensor that has high sensitivity and speed to record at short exposures and fast frame rates, like ImageStream [31,44,45,46]. However, such a camera is often expensive for practical research or clinical uses, especially when multiple cameras are required to capture multiple fluorescence channels, and its data transfer can be complicated, hampering the integration with real-time high-throughput cell sorters. Considering this challenge of implementing high-sensitive cameras for fluorescence imaging, using relatively inexpensive CMOS cameras for brightfield imaging is a more reasonable approach for commercialization [47,48]. On the other hand, another key approach that has recently emerged in IFC to circumvent using cameras is single-pixel imaging, which we focus on in this section [7,49,50,51,52].

Single-pixel imaging does not require a camera or any type of sensor array, and instead, it is able to reconstruct the image from the temporal signal, as if a camera were used. Laser-scanning microscopy is an example that may be more familiar, where each pixel in an image is acquired sequentially and is tiled one by one to construct a single image. The issue with typical laser-scanning microscopy is that the scanning speed is limited by the mechanical scanner, which is around 20 kHz for the fastest commercially available resonant scanners [53]. Considering that imaging of a cell requires multiple scans to create a single image, the imaging throughput with a mechanical scanner will be less than 1000 cells per second, which is slower than ImageStream. Therefore, to achieve a higher throughput, technologies to perform single-pixel imaging without mechanical scanning have been invented.

Another key concept in IFC is utilizing the translation of the cell caused by the flow for the acquisition of the image. This will be referred to as optofluidic imaging in this review, although optofluidics refers to a broader concept of utilizing fluidics for optics. The most common optofluidic approach for IFC is utilizing the flow for scanning the cell in the direction of the flow while acquiring the other dimension(s) with optical scanning, sensor arrays, or single-pixel imaging, reducing the dimension that the optics need to span [50,51]. This is especially powerful when performing single-pixel imaging because it reduces the complexity of adding another scanning method in the orthogonal axis to the fast-scanning axis. A less common, but still powerful, optofluidic approach is to utilize the flow to move a cell through a structured illumination to convert the spatial information of the cell to a temporal signal [7,52]. With this approach, no scanning of the illumination is required, and therefore, the imaging speed will be limited by the velocity of the cell and the detector bandwidth rather than the scanning speed. Examples of this approach will be shown later in this section.

#### 2.2.1. Optical Time-Stretch Microscopy

**Figure 2:**
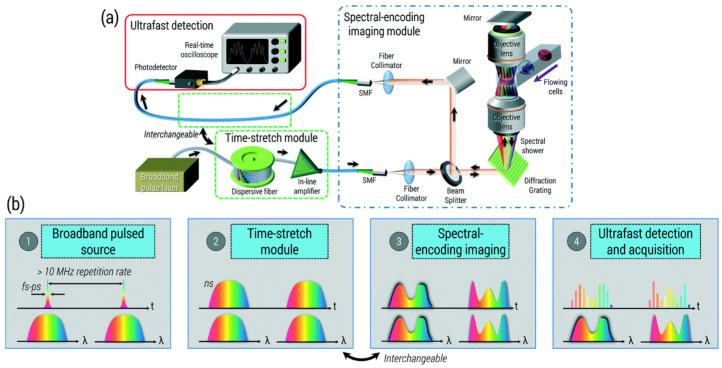
Optical time-stretch (OTS) microscopy. (**a**) Overall schematic of the optical setup. (**b**) The change in temporal and spectral components of optical pulses as they go through each module in (**a**). The original femtosecond pulse is stretched temporally using chromatic dispersion through a long fiber. Then, each spectral component is mapped to a different spatial position on the channel where the cells flow, and the light attenuation at each spatial position is encoded onto the spectrum, which also corresponds to the temporal pulse shape. In the end, an ultrafast detector captures the temporal pulse shape, which represents the spatial profile at the cross-section of the channel. Reproduced with the permission of the Royal Society of Chemistry from [55].

One of the key inventions for IFC is optical time-stretch (OTS) microscopy [49,50,54,55]. This technology utilizes broadband near-infrared (NIR) femtosecond pulse lasers to perform ultrafast scan-less imaging (Figure 2). NIR femtosecond pulse lasers consist of wide spectral components ranging to tens of nanometers, and by splitting this spectral component spatially, typically with a diffraction grating, it is able to perform imaging of different spatial positions with different spectral components. Then, to temporally read out these spectral components, the original pulse is dispersed temporally with a long fiber, in which the speed of light is different at different wavelengths. The temporally dispersed pulse is detected in the end with a high-speed photodetector to obtain an image as if the laser was scanned across the imaging plane.

The major advantage of OTS is its extremely high imaging speed, typically about 40 MHz for line imaging, which is three orders faster than mechanical scanning. Because of this, it can easily capture blur-free images of cells that flow at 10 m/s, and the limitation comes from the ability to flow the cells at higher velocities rather than the imaging capability. Using OTS, high-throughput IFC at 100,000 cells/s was performed to demonstrate detection of spiked cancer cells from 10 million blood cells at a very low false positive rate [50]. Furthermore, because it performs imaging with a coherent light, besides the original bright-field imaging, phase contrast and quantitative phase imaging were realized to capture refractive index distributions within the cell [54,56,57].

Still, one of the major disadvantages of OTS is that it cannot be used for fluorescence imaging. This is because OTS relies on the spectral component of the pulse laser to encode the spatial information, but this correlation will disappear when the laser is used as an excitation and converted to fluorescence emission. Nevertheless, OTS can be combined with fluorescence signal measurement similar to conventional flow cytometry to perform analysis based on fluorophore biomarkers [58]. Yet, many valuable information lies inside subcellular organelles and structures that require intracellular fluorescent imaging.

#### 2.2.2. Fluorescence Imaging Using Radiofrequency-Tagged Emission

**Figure 3:**
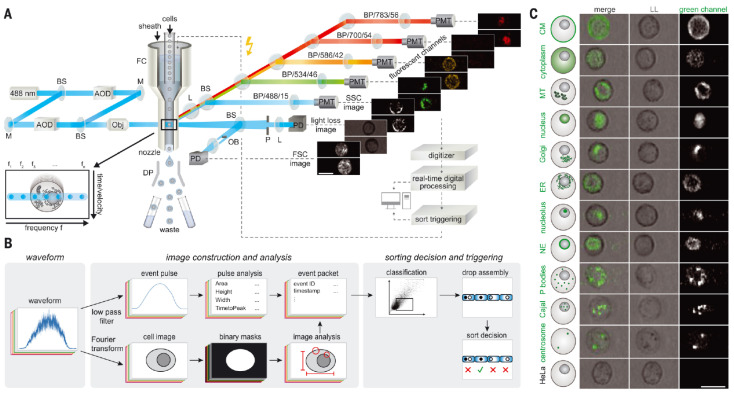
Cell sorter with fluorescence imaging using radiofrequency-tagged emission (FIRE). (**A**) Schematic of FIRE and cell sorting. Different frequency component-modulated beams are directed to different horizontal positions of cell. Resulting fluorescence will also have corresponding modulation and this can be decoded by signal processing. (**B**) Real-time signal processing, image analysis, and evaluation for cell sorting. (**C**) Example cell images of different organelles obtained with FIRE. Scale bar, 20 µm. From [9]. Reprinted with permission from AAAS.

Fluorescence imaging using radiofrequency-tagged emission (FIRE) is a method that was developed to enable fluorescence imaging at high-speed conditions of IFC [51]. This method is another single-pixel imaging that encodes the spatial information onto the frequency domain of the light signal. The excitation beam is split into multiple beams having different radiofrequency modulation and then directed to different spatial positions in the channel (Figure 3A). Because this modulation is also transferred to the fluorescence signal, by collecting the temporal fluorescence signal and performing a Fourier transform to convert it to the frequency domain, a fluorescence image can be created (Figure 3B). The modulation that can be applied is in the order of tens of MHz, and for a moderate resolution, the line scan frequency can be in the order of hundreds of kHz. With this, blur-free imaging of cells flowing at a velocity of 1 m/s was achieved (Figure 3C).

Due to the capability of fluorescence imaging at a high flow velocity, FIRE has been implemented for fluorescence-image-activated cell sorters (FICSs) [8,9]. Although the largest obstacle for FIRE to be used for FICSs was the real-time computation for recovering the image via Fourier transform, this was realized by either lengthening the channel to extend the duration for interrogation or using FPGAs to process the signal at a high speed. In its recent implementation, the sorting throughput reached up to 15,000 events/s, which is comparable to conventional flow cytometry cell sorters [9]. This sorter enabled enriching cells in a particular mitotic stage without requiring toxic chemical blockers to synchronize their cell cycle. It was also demonstrated that FICS can be used for speeding up pooled genetic screens of fluorescence image phenotypes, which previously required microscopy measurements.

#### 2.2.3. IFC with Spatial–Temporal Transformation

**Figure 4:**
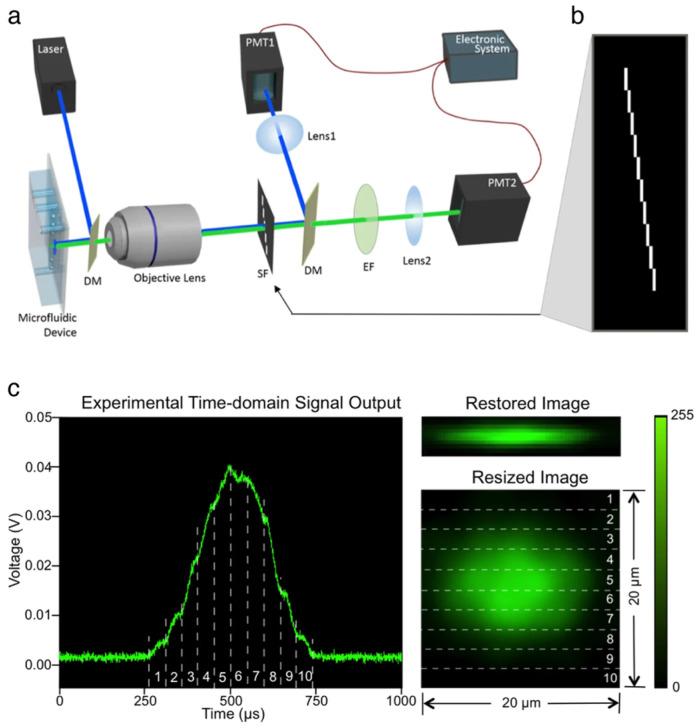
IFC with spatial–temporal transformation. (**a**) Optical schematic. (**b**) A spatial filter used to map the spatial information of a cell onto the temporal signal. Each slit in the spatial filter obtains a vertical profile of a horizontal position of the cell. (**c**) Raw waveform from a single cell and its reconstructed image. Each number in the plot corresponds to a profile obtained with a single slit on the spatial filter. These are then stacked to reconstruct the 2D image. Adapted from [52].

Another single-pixel fluorescence imaging technique for IFC is a spatial–temporal transformation method developed by Han and Lo [52]. This uses a simple optical setup for converting the spatial information of the cell to the temporal information of the fluorescence signal with an optofluidic approach (Figure 4a). A spatial filter with a pattern as in Figure 4b is used in the detection path to detect the fluorescence signal from a column of pixels one by one as the cell flows through, and by stacking the temporal signal from each column, a fluorescence image of the cell can be obtained (Figure 4c). Using this, imaging of cells at 0.2 m/s was achieved. Although this system may not be as fast as the previously mentioned technologies, it is highly versatile because of its simplicity, and can be expanded to bright-field imaging [59], 3D imaging [10], and hyperspectral imaging [60]. Furthermore, it is also compatible with image-activated cell sorting [36].

On the other hand, there are several limitations to this spatial–temporal transformation method. One is that when a cell enters the imaging region while another cell is still in it, the signals overlap, and an image cannot be restored. Because the pattern of the spatial filter spans to the direction of the flow, it requires sufficient spacing between the cells and limits the possible throughput. Another major drawback is that the resolution is limited to 1–2 µm by the aperture size of the spatial filter, which cannot be reduced to the best of our knowledge because that will cause the length of the spatial filter to further increase. Besides these, one limitation of the original method was that the light-power efficiency was low because the spatial filter cuts off the fluorescence light from the cell, but this can be resolved by creating the pattern on the excitation light with a spatial-light modulator or a diffractive optical element [60].

#### 2.2.4. Ghost Cytometry

**Figure 5:**
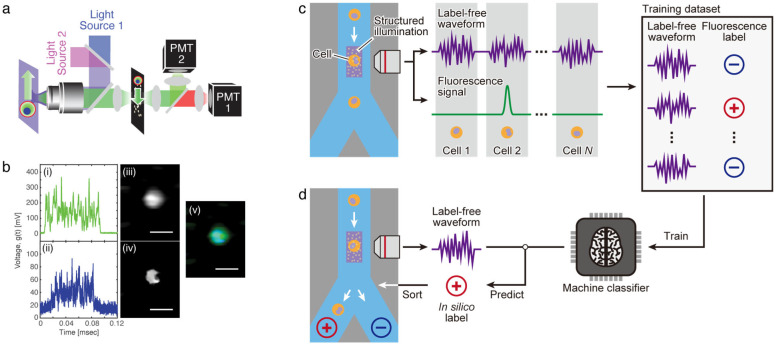
Ghost cytometry. (**a**) Optical schematic for fluorescence ghost cytometry. A spatial filter before the detectors (PMTs) is used in this schematic, but a structured illumination can also be used similar to (**c**). (**b**) Raw waveforms and reconstructed images from a single flowing cell. From the fluorescence signals from the cytoplasm (i) and nucleus (ii), images (iii) and (iv) were reconstructed, respectively. (v) is an overlay of (iii) and (iv). Scale bars, 20 µm. (**c**,**d**) Cell sorting with in silico-labeled ghost cytometry. The label-free waveform containing image information is obtained simultaneously with the fluorescence label for training the machine-learning classifier (**c**). Then, the classifier can predict the label without using fluorescence from the label-free waveform and perform sorting based on this prediction (**d**). (**a**,**b**) are from [7]; adapted with permission from AAAS. (**c**,**d**) are adapted from [64].

One particular IFC technology that is unlike any other method is ghost cytometry [7]. Ghost cytometry utilizes a compressive sensing concept called ghost imaging [61] and performs single-shot single-pixel imaging with optofluidics. A predefined, random sparse pattern of illumination encodes the spatial information of a cell as a temporal signal while the cell moves through the patterned illumination (Figure 5a). From the temporal signal and the predefined random illumination pattern, multicolor fluorescence images of the flowing cell are recovered computationally (Figure 5b).

The major uniqueness of ghost cytometry is that it compressively acquires image information of cells and performs their classification and interrogation without constructing an actual image. The compressive sensing enables the same amount of spatial information to be fit into a shorter temporal signal and is therefore able to encode finer information compared to a linear pattern. Because the temporal signals are the sources of the reconstructed image, an image-construction-free classification can be performed directly with the signals. As a result, this allows for bypassing the computational time required for image reconstruction and for realizing an approach for increasing throughput while maintaining accuracy for both analysis and sorting purposes [7,62,63,64].

A key to this image-construction-free classification is developing a machine learning model that is trained based on prior-acquired data. This can be performed with both supervised and unsupervised training. Supervised learning is effective when the classification labels are available for each cell. For instance, by training the model using labels defined with antibody markers (Figure 5c), it will be able to classify cells that have not been stained with those markers, by inferring their antibody label from the 1D temporal imaging signals (Figure 5d). This inference of molecular labels, called in silico labeling, can be applied to many applications [65]. Ghost cytometry can perform in silico labeling with modalities that do not require fluorescence staining, and such capability has the potential for medical applications such as leukemia detection, cell therapy manufacturing, and large-scale pooled genetic screens, where users want to avoid the staining due to the required time and cost or potential staining-associated side effects [32,64,66,67,68]. On the other hand, an unsupervised approach can be highly versatile and valuable especially when appropriate classification labels are not available, such as when addressing a phenomenon or disease that has not yet been well characterized or understood. In this case, the multiparametric imaging signals can be subject to dimensional reduction methods, and from the clusterized data, a model can be developed to classify and sort the cells of interest.

### 2.3. Three-Dimensional IFC

Although many 2D IFC technologies have proved to be powerful in analyzing and sorting cells based on their morphological information, there are often cases where the loss of information in the depth direction of the image substantially affects the analysis, and thus, 3D imaging becomes necessary. For example, many subcellular structures are organized in a 3D fashion, and it is sometimes critical to resolve them in 3D. Accurate identification of co-localization events inside the cell also requires 3D imaging to confirm whether features are actually coinciding or appearing to be overlapping because of the orientation. Similarly, 2D imaging can result in occlusions of features, which results in misidentification. Considering that the original motivation for IFC was to reduce the false-positive and false-negative rates during analysis, developing 3D IFC technologies becomes the straightforward approach to achieve higher sensitivity and specificity.

However, performing 3D imaging of cells while they are in flow becomes even more challenging than 2D imaging. Attempts have been made to perform IFC by spinning disk confocal microscopy and light-sheet microscopy methods [69,70,71], but the throughputs were less than 10 cells/s, which are too slow for population screening, and moreover, did not show an advantage over automated microscopy with well plates or dishes. In addition to the speed, high resolution also becomes necessary to accurately resolve the structures of interest in the 3D space: subcellular structures for single cells and individual cells or smaller for clusters of cells such as spheroids and organoids. If it does not have sufficient spatial resolution, 2D IFC would be more beneficial because it has higher throughputs. For these reasons, technologies that meet both criteria of having high throughput while having sufficient subcellular resolution will be discussed in this review.

#### 2.3.1. Camera-Less 3D IFC

**Figure 6:**
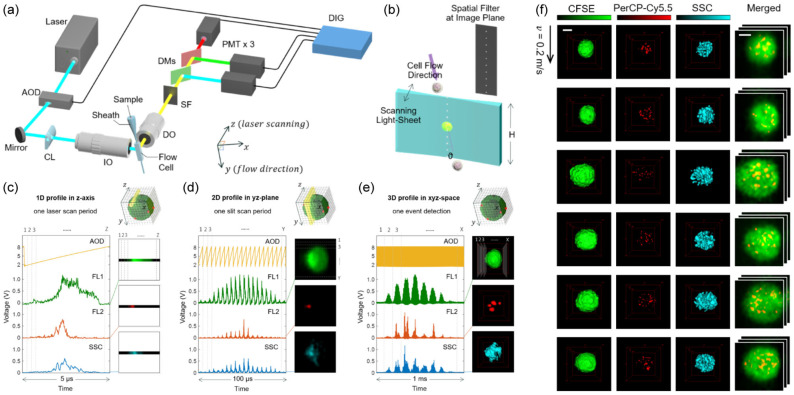
Camera-less 3D IFC. (**a**) Optical schematic. (**b**) Orientation of the spatial filter and light sheet in relation to the cell flow. (**c**–**e**) 3D acquisition process. (**c**) First, the light sheet is scanned in the z-axis direction with the AOD to acquire the cell profile in the z-axis direction. (**d**) Then, the cell translates in the y-axis direction due to the flow, and a single slit of the spatial filter acquires the cell profile in the y-axis direction. (**e**) Finally, different slits on the spatial filter acquire different profiles in the x-axis direction to consequently form a 3D image. (**f**) Obtained 3D images of CMK3 cells stained with CFSE and γH2AX antibody-conjugated with PerCP/Cy5.5. Scale bars, 5 µm. Adapted with permission from [10]; © Optical Society of America.

The first successful implementation of 3D IFC at high throughput and with subcellular resolution was the 3D extension of the spatial–temporal transformation by Han and Lo (Figure 6) [10]. This camera-less 3D IFC uses a similar spatial filter in the detection path as the 2D spatial–temporal transformation but changes the excitation beam so that it is scanned in the depth direction (Figure 6b). The excitation beam is scanned at high speed using an acousto-optic deflector (AOD) so that, for each pixel of its 2D counterpart, the whole depth axis is scanned (Figure 6c). Then, in a similar scheme as the 2D version, the other two dimensions are acquired to obtain a 3D volume of a cell (Figure 6d,e). This enabled 3D IFC at a flow velocity of 0.2 m/s, similar to its 2D counterpart, while achieving spatial resolution of about 2 µm, and demonstrated that the amount of DNA damage inside the cell can be quantified, which would be difficult with 2D IFC because of occlusions of fluorescence spots [10]. This 3D IFC can be combined with 3D convolutional neural networks to classify cells at higher accuracy than what is possible with 2D IFC [72].

Still, there are limitations to this camera-less 3D IFC that are similar to its 2D counterpart. One major drawback is that it is difficult to improve the resolution. Like the 2D version, the number of pixels in the lateral direction is defined by the apertures in the spatial filter and is difficult to extend. In addition to this, because each aperture is required to collect the fluorescence from different depth positions during the z-axis scanning, it is difficult to increase the NA of the detection. The second drawback is the throughput, which holds the same as the 2D version. Even though the theoretical throughput is 500–1000 cells/s assuming that the cells will be separated by the length of the spatial filter pattern [73], typically cells are distributed in flow according to Poisson statistics. Therefore, the practical throughput can be estimated to be lower, depending on whether the purity or the quantity of the data is prioritized. The third limitation is, again, the power efficiency, which limits the ability to image at higher flow velocities that require shorter exposure times.

#### 2.3.2. Parallel 3D IFC

Unlike 2D IFC, when it comes to 3D imaging, using a high-speed camera can become faster than single-pixel imaging because it eliminates the number of dimensions that needs to be scanned. Furthermore, because each pixel on a camera array operates parallelly, at equal pixel acquisition rates to single-pixel imaging, they can have longer exposure times to increase the signal-to-noise ratio. For these reasons, light-sheet microscopy has become popular in many applications where high-speed volumetric imaging is required [74,75,76,77,78]. Such advantage also holds true for 3D IFC, and therefore, by utilizing the full potential of light-sheet imaging, high-throughput 3D IFC becomes possible. Still, the frame rate of high-speed fluorescence cameras such as sCMOS cameras was limited to around 1000 frames/s at a reasonable active area, leading to a volume rate of less than an order of magnitude slower, and because of this, high-throughput 3D IFC was difficult by imaging each cell one by one.

**Figure 7:**
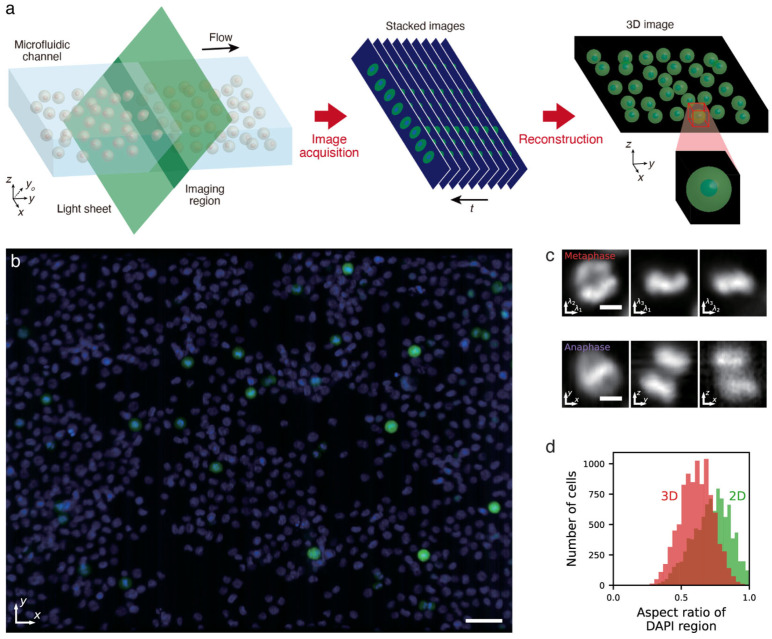
Parallel 3D IFC. (**a**) Simplified schematic. Oblique-plane imaging continuously acquires a diagonal cross-section of a rectangular channel while the cells flow through. This time series is stacked horizontally to reconstruct a 3D image of the whole population. (**b**) xy cross-sectional view of a K562 cell population stained with DAPI (blue) and MPM-2 anti-mitotic protein antibody (green). Scale bar, 50 µm. (**c**) Cross-sectional DAPI images of cells in metaphase and anaphase. Scale bars, 5 µm. (**d**) A 3D and 2D morphology analysis of 10,814 mitotic cells within 408,937 cells imaged with 3D IFC. Adapted from [11].

To overcome this limitation, parallel 3D IFC was developed to perform parallel acquisition of multiple cells with a single-objective-lens light-sheet microscopy technique called oblique-plane microscopy (Figure 7) [11,79,80]. Furthermore, this parallel acquisition of cells in flow is sustained by fluidic control of cells using acoustic waves so that all the cells flow at the same flow velocity to acquire their 3D volume in an optofluidic way. As a result, parallel 3D IFC was able to harvest the potential of light-sheet imaging to achieve high-throughput 3D IFC at a throughput of over 2000 cells/s, the fastest to our knowledge, while having a spatial resolution of about 1 µm. With such throughput, this technology was able to collect 3D cell images of 400,000 cells in a total acquisition time of less than 5 min and perform quantification of cells based on 3D subcellular structures (Figure 7d). This demonstrated that 3D IFC is capable of large-population cell analysis that is comparable to conventional flow cytometry and 2D IFC.

Such high-throughput 3D IFC achieves what was difficult with 2D IFC or 3D microscopy. For example, detecting morphological differences in organelles of small subpopulations requires large populational screening to capture a sufficient quantity of the cells in the subpopulation and, at the same time, requires 3D imaging to recognize the difference accurately. In particular, detection of mitotic abnormalities involves the chromosome being visualized in mitotic cells, which only exists in a fractional amount [27,81]. As seen in Figure 7c, parallel 3D IFC can identify the chromosomal structure of mitotic cells, which would be overlooked by only 2D imaging [81]. Furthermore, its scalability allows for capturing more than 10,000 cells in mitosis (Figure 7d), potentially leading to the future development of machine-learning models for detecting atypical mitotic figures.

The biggest advantage of parallel 3D IFC is that all the cells that pass inside the imaging region can be imaged without any occlusions and can be resolved. Therefore, with careful design of the flow channel, all cells that flow through can be imaged regardless of the concentration. This is advantageous for imaging clusters of cells, which exist in actual blood samples, without missing a cell, which is not possible with 2D IFC [82]. On the other hand, the biggest disadvantage of parallel 3D IFC is that because the cells flow parallelly, it is difficult to combine with a high-throughput cell sorting mechanism, which typically requires the cells to be processed one at a time.

#### 2.3.3. FLITS

**Figure 8:**
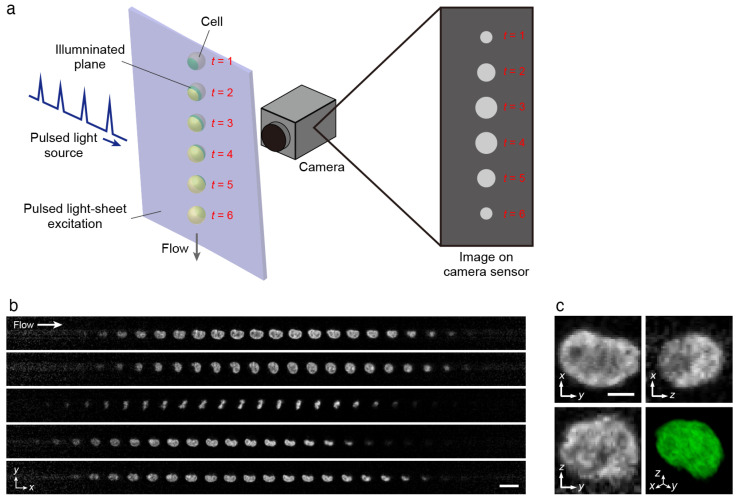
High-flow-velocity 3D IFC with FLITS. (**a**) Optical schematic. A light-sheet excitation created with a pulsed light source is oriented at a slight angle to the direction of the cell flow. As the cell flows through the light-sheet excitation, different planes of the cell are illuminated, each with a single pulse at different time points. These are all collectively acquired with a single exposure on the camera that is placed in the orthogonal direction to the light sheet so that each illuminated plane will be mapped on different positions of the sensor. The imaged planes in a single frame are stacked to reconstruct a 3D image of a cell. (**b**) Example acquired images on the camera of K562 cells stained with SYTOX Green flowing at over 11 m/s. Scale bar, 20 µm. (**c**) Cross-sectional views and isometric view from a 3D reconstructed image of a cell from the top image in (**b**). Scale bar, 5 µm. Adapted with permission from [83]; © Optica Publishing Group.

In contrast to parallel 3D IFC, a technology called optofluidic light-sheet imaging with transformation of spatial information (FLITS) enables 3D imaging of cells flowing one by one at high speeds [83]. This technology uses a stroboscopic light-sheet illumination that is placed slightly at an angle to the channel flow direction so that when a cell flows through this region, each cross-section of the cell will be illuminated with each pulse of the stroboscopic illumination (Figure 8a). Then, a single exposure of a camera collects all these cross-sections to produce a 3D image of the whole cell. As a result, FLITS can image cells at a flow velocity of over 10 m/s (Figure 8b,c), which is the fastest 3D IFC comparable to the fastest 2D fluorescence IFC in terms of flow velocity, and this speed is only limited by the fluorescence lifetime of the fluorophore. It has been demonstrated that even at this velocity, blur-free images of subcellular structures can be obtained with sub-micrometer isotropic resolution.

Because FLITS can capture 3D images of cells one by one in a sequence, it has the potential to be implemented with cell sorting. The theoretical throughput of FLITS is about 1200 cells/s, corresponding to the frame rate of current sCMOS cameras, and can be higher with development of even faster cameras. Also, compared to other methods that require spatial–temporal transformation, the exposure time is much shorter than the camera refresh time, so there is less possibility of data abortion due to cells overlapping in the imaging region by Poisson distribution, and therefore, the practically possible throughput will become close to the theoretical throughput. For these reasons, 3D-image-based cell sorting at over 1000 cells/s can become possible with FLITS.

#### 2.3.4. Light-Field Flow Cytometry

**Figure 9:**
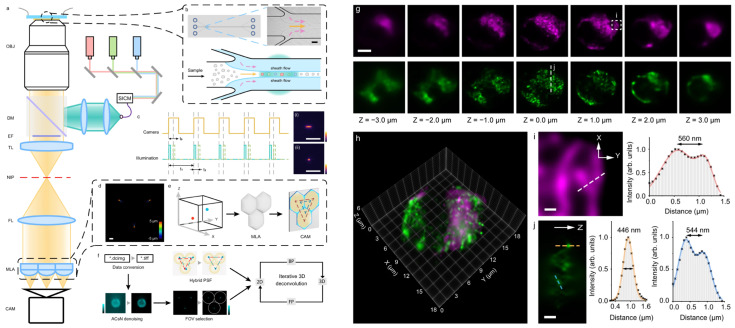
Light-field flow cytometry. (**a**–**f**) Overall schematic. The image created at the native image plane (NIP) is relayed to the camera while having a microlens array (MLA) before the camera instead of a lens (**a**). Because each microlens in the array creates on the camera an image of the cell from different view angles, a 3D image can be computationally reconstructed from those views. (**g**–**j**) 3D reconstructed images of mitochondria (magenta) and peroxisome (green) of HeLa cells. (**g**) Reconstructed images of different planes. Scale bar, 5 µm. (**h**) Merged 3D image. (**i**,**j**) Zoomed-in images. Scale bars, 500 nm. Adapted from [84].

Another interesting camera-based 3D IFC technology is light-field flow cytometry (Figure 9) [84]. This uses an imaging technique called Fourier light-field microscopy (LFM), which captures spatio-angular information of light on a camera from which a 3D image can be reconstructed computationally [85,86]. With this technique, fluorescence imaging is performed with a setup similar to epifluorescence microscopy, except the imaging plane after the tube lens is passed through another lens, and in the focus of this lens (called the Fourier plane), a microlens array is placed that focuses the image onto a camera (Figure 9a). Since Fourier LFM requires only a single exposure to capture a 3D image of a cell, it can have the potential to enable 3D IFC at high flow velocities and throughputs.

Still, Fourier LFM suffers from non-uniform spatial resolution across different depths, and the resolution decays the farther the reconstructed plane is away from the focus plane [86]. This is especially prominent at high NAs where the resolution at the focus can become several hundred nanometers, but the resolution rapidly decays several micrometers away from the focus (Figure 9g). Therefore, it is uncertain whether a cell with a diameter of over 10 µm can be imaged with sufficient resolution across depths with the demonstrated conditions. Nevertheless, light-field flow cytometry can be a useful technique for 3D IFC if the configuration is sufficiently adjusted depending on the situation.

## 3. Discussion

### 3.1. Resolution and Throughput

In IFC, there is always a tradeoff between the amount of data obtained per cell versus the cell throughput. In other words, 3D IFC will have lower throughput than 2D IFC, and higher resolution IFC will have lower throughput than lower resolution IFC (Table 1). Even within the same method, the resolution and the throughput can be adapted based on the analysis of interest, i.e., for an analysis that requires obtaining finer features of the cell, the resolution can be increased by sacrificing the throughput, and for an analysis that does not require such resolution, the resolution can be traded for higher throughput. This is similar to switching an objective lens on a standard microscope to change the magnification and, for most IFC technologies, this can actually be changed by switching the objective lens that is used.

**Figure 10:**
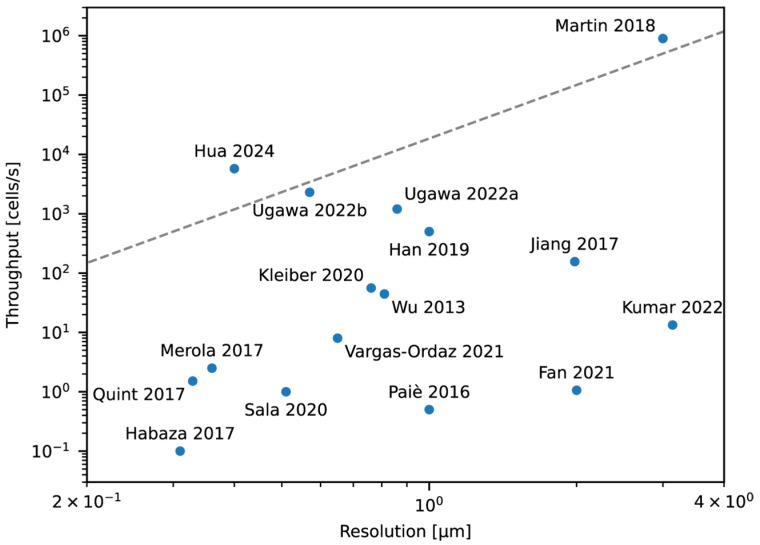
Comparison of 3D IFC technologies [10,11,69,70,71,83,84,88,89,90,91,92,93,94,95,96]. Dashed line shows the theoretical limit with current sCMOS cameras. Martin, 2018 [89] uses a linear PMT array and is able to surpass this limit. Hua, 2024 [84] does not estimate the throughput based on the frame rate of the camera and therefore exceeds this limit. Plotted values are based on the best reported throughput and resolution in the dimension that has the smallest value if provided or an estimate based on the reported measurement parameters. Ugawa, 2022a and Ugawa, 2022b correspond to references [83] and [11], respectively.

Therefore, to compare the speed between different methods, it is sometimes useful to compare based on the resolution (or the number of pixels or voxels) × throughput product as in Figure 10. This is ultimately limited by the data transfer rate of the detection system unless there is a fundamental limit elsewhere. The closer this product is to the data transfer rate, shown as a dotted line in Figure 10, the more efficient the method is in utilizing the speed of the detection system. Because this data transfer rate is dependent on the type of detector or camera that is used, advancements in detector or camera technology will extend the resolution × throughput product in the future. Thus, the values that are given in this review for a particular method will not be permanent and are anticipated to improve in future implementations.

### 3.2. Prospects for IFC

With the IFC technologies reaching comparable throughputs to conventional flow cytometry, more of the challenge now lies in the analysis of the data that is acquired with IFC. For 2D and, especially, 3D IFC, cell analysis methods are still in the course of development, and there is no standard practice that is similar to conventional flow cytometry. The most common strategy is to use a range of feature metrics such as area, diameter, eccentricity, etc., and draw gates in scatter plots or dimensionally reduced plots similar to fluorescence scatter plots in conventional flow cytometry. This is a well-controlled analysis system that can be resistant to artifacts, but on the other hand, may not be able to extract information that is difficult to explicitly describe as a feature. In contrast, a machine-learning-based analysis such as using convolutional neural networks can be used to extract unknown features that lie in the images [38,72,97]. However, in such cases, it is critical to train the model appropriately so that it generalizes to avoid overfitting experimental artifacts. At the moment, users will have to choose the type of analysis suitable for their purpose, which may create a hurdle for new IFC users and add biases to the analysis. Still, as more users are involved, it is anticipated that collected knowledge will be accumulated to provide standardized practice for choosing and tuning a model.

Related to the analysis, the standardization and sharing of IFC datasets will also be important, as is also the case in any other flow cytometry or microscopy systems. Currently, the IFC datasets obtained with ImageStream are often shared, but those obtained with other commercial IFC systems will start to be shared in the future. Organizing a standard for multiple platforms will become useful in the future because, if machine learning-based analysis becomes applicable across different systems, larger datasets can be created for potentially more reliable, generalized, and accurate analyses.

As a more technical prospect, scaling IFC so that it can be applicable to groups of cells such as spheroids and organoids will be one direction that will have significant importance [82,88]. For this purpose, 3D imaging will be necessary to image cells that are behind each other. Furthermore, not only the morphology of a cell becomes important, but also the spatial distribution and connection between cells become highly important features. Still, this is not a simple task with the current IFC technology because controlling a large cluster of cells in flow is challenging, as is imaging through multiple layers of cells without degrading the resolution.

In addition, higher-dimensional imaging, which has additional dimensions beyond the spatial dimensions, is becoming more widespread in the microscopy field and is expected to migrate toward IFC. There are already some reports on hyperspectral fluorescence imaging, which adds the dimension of the fluorescence spectrum in 2D IFC [60]. Another spectroscopic imaging method that can be used for IFC is Raman imaging. Although spontaneous Raman scattering is typically weak for high-speed imaging, coherent Raman scattering methods such as stimulated Raman scattering can enhance the signal for imaging fast enough for IFC [98]. A more recently trending imaging technique is fluorescence-lifetime imaging [99], which can also be expected to be applicable for IFC as it is capable of high-speed imaging. A further trending imaging technique that is compatible with high-speed imaging is quantitative phase imaging, which obtains the refractive index profile of the cell [57,94,100,101]. While quantitative phase imaging does not directly obtain biomolecular information, combining it with multimodal data through machine learning can enable it to decipher the intracellular structure inside a cell. The major drawback is the computational resources necessary for reconstructing the images of thousands of cells in a practical amount of time, but this may be circumvented with machine learning or other methods. Lastly, perhaps one of the most technically difficult additions for IFC is time-lapse imaging because individual cells are difficult to track after imaging in flow. There has been a reported technology for time-lapse flow cytometry measurements using laser-emitting semiconductor particles [102], but these particles would most likely cause interference with the cell images if implemented for IFC. On the other hand, image-based barcoding methods to track microparticles and droplets have also been developed [103,104,105], and utilizing these methods may provide a solution for time-lapse IFC.

## 4. Conclusions

With the advance of optical and information technologies, IFC has transformed over the last few decades from a mere concept of combining the advantages of flow cytometry and microscopy into an accessible tool for high-throughput, high-content single-cell analysis. Indeed, 2D IFC technologies such as those that have been discussed have become mature enough to be commercialized or are on their way to commercialization. On the other hand, 3D and higher-dimensional IFC technologies require further development regarding efficient data processing and key applications. Moreover, the greater challenge now lies in comprehending and utilizing the vast data obtained by IFC. We expect this challenge to be addressed as IFC becomes more prevalent in various fields, in close coordination with efforts to cultivate collective knowledge about data analysis. As IFC technology and the subsequent knowledge generation pipeline becomes more accessible, its impact on clinical diagnostics, the biomedical industry, and life science research will expand, establishing its role as a cornerstone of biomedical research and applications.

## Figures and Tables

**Table 1:** Summary of 2D and 3D IFC technologies. Values are based on representative implementations.

Technology	Imaging Dimensions	Fluorescence Imaging	Resolution (µm) *^1^	Throughput (Cells/s)	Flow Velocity	Cell Sorting *^2^	Reference
ImageStream	2D	Yes	0.4 *^3^	300 *^4^	No data	No	[87]
OTS microscopy	2D	No	1.4	100,000 *^5^	10 m/s	No	[50]
FIRE	2D	Yes	2	15,000	1 m/s	Yes	[9]
Spatial–temporal transformation	2D	Yes	0.6 *^3^	1000 *^5^	0.2 m/s	Yes	[52]
Ghost cytometry	2D	Yes	<1	10,000 *^5^	10 m/s	Yes	[7]
Camera-less 3D IFC	3D	Yes	1	500 *^5^	0.2 m/s	No	[10]
Parallel 3D IFC	3D	Yes	0.57	2300	0.5 mm/s	No	[11]
FLITS	3D	Yes	0.86	1200 *^5^	11.4 m/s	No	[83]
Light-field flow cytometry	3D	Yes	0.4	5750 *^5^*^6^	115 mm/s	No	[84]

*^1^ Resolution in the dimension that has the smallest value. *^2^ Considers only currently reported implementation. *^3^ Estimated from the NA of the objective used. *^4^ Throughput can go up to 5000 cells/s by changing resolution. *^5^ Theoretical throughput assuming equal spacing between cells. *^6^ Does not consider the frame rate of the camera (see also Figure 10).

## Data Availability

Not applicable.
